# Correction: Yin et al. Angiogenesis–Browning Interplay Mediated by Asprosin-Deficiency Contributes to Weight Loss in Mice with Obesity. *Int. J. Mol. Sci.* 2022, *23*, 16166

**DOI:** 10.3390/ijms26062499

**Published:** 2025-03-11

**Authors:** Tingting Yin, Sheng Chen, Guohua Zeng, Wanwan Yuan, Yanli Lu, Yanan Zhang, Qianqian Huang, Xiaowei Xiong, Baohua Xu, Qiren Huang

**Affiliations:** 1 Key Provincial Laboratory of Basic Pharmacology, Nanchang University, Nanchang 330006, China; tingtingyin007@163.com (T.Y.); chensheng@email.ncu.edu.cn (S.C.); guohuazeng@ncu.edu.cn (G.Z.); wanwan_yuan@163.com (W.Y.); yllu@email.ncu.edu.cn (Y.L.); zyn21221452@163.com (Y.Z.); qianqianhuang@email.ncu.edu.cn (Q.H.); xwx9309@email.ncu.edu.cn (X.X.); 2 Department of Pharmacology, School of Pharmacy, Nanchang University, Nanchang 330006, China; 3 Jiangxi Province Key Laboratory of Laboratory Animal, Nanchang 330006, China

In the original publication [1], “asprosin-knockout” is somewhat inappropriate, but we indeed generated ASP deficiency in adipose tissue of mice, evidenced by the attachment titled “Supplementary Materials”. Therefore, “Asprosin-Knockout”, “*ASP*-knockout”, “*ASP*-CKO”, and “*ASP^−/−^*” in the original publication [1] were revised to “Asprosin-Deficiency”, “*ASP-deficiency*”, “*ASP-deficiency*”, and “*ASP-def*” throughout the paper, including the corresponding Figures 1–4. The authors state that the scientific conclusions are unaffected. This correction was approved by the academic editor. The original publication has also been updated.

The correct Figure 1 and Figure 2 appear below.

## Figures and Tables

**Figure 1:**
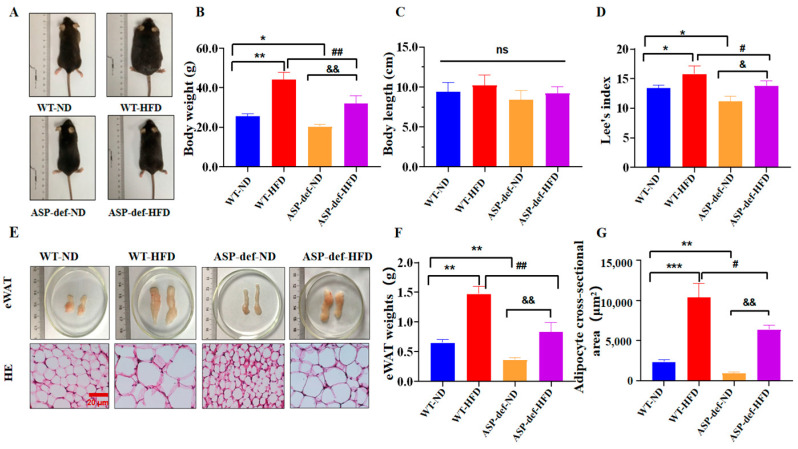
*ASP-deficiency* alleviates mouse obesity induced by HFD. Representative photographs (**A**) of C57 BL/6 mice. Body weight (**B**), length (**C**), and Lee’s index (**D**) were measured and calculated. Representative photographs (**E**) and weights (**F**) from eWAT in mice. Representative HE-staining images from eWAT (**E**) and average diameters of adipocytes from eWAT. (**G**) Data are presented as means ± SEM from 3 independent experiments (*n* = 3). Two-way ANOVAs were performed, followed by unpaired two-tailed Student’s *t*-tests. * *p* < 0.05, ** *p* < 0.01, *** *p* < 0.001 vs. WT-ND group; ^#^ *p* < 0.05, ^##^ *p* < 0.01 vs. WT-HFD group; ^&^ *p* < 0.05, ^&&^ *p* < 0.01 vs. *ASP-def*-ND group; ns = no significance. WT-ND: wild-type mice fed with normal diet; WT-HFD: wild-type mice fed with high-fat diet; *ASP-def*-ND: *ASP-deficient* mice fed with normal diet; *ASP-def*-HFD: *ASP-deficient* mice fed with high-fat diet; eWAT: epididymal white adipose tissue. Scale bars in the images of HE staining in (**E**) 20 µm. *ASP*: asprosin, HE staining: hematoxylin–eosin staining.

**Figure 2:**
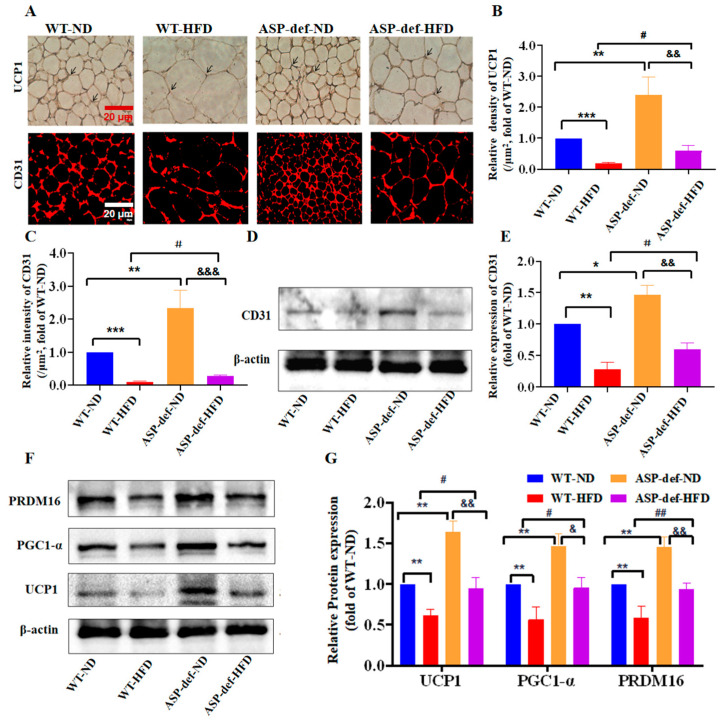
*ASP-deficiency* promotes angiogenesis and browning of white adipose in vivo. Representative images (**A**) of immunohistochemistry and immunofluorescence. Relative expression levels in situ of UCP1 (**B**) and CD31 (**C**) in eWAT normalized by adipocyte size. Expression levels of CD31 in eWAT by Western blot (**D**,**E**). Expression levels of the browning-related proteins, including PRDM16, PGC-1α, and UCP-1, in eWAT (**F**,**G**). Data are presented as means ± SEM from three independent experiments (*n* = 3). Two-way ANOVAs were performed followed by unpaired two-tailed Student’s *t*-tests. * *p* < 0.05, ** *p* < 0.01, *** *p* < 0.001 vs. the WT-ND group; ^#^ *p* < 0.05, ^##^ *p* < 0.01 vs. the WT-HFD group; ^&^ *p* < 0.05, ^&&^ *p* < 0.01, ^&&&^ *p* < 0.001 vs. the *ASP-def*-ND group. WT-ND: wild-type mice fed with normal diet; WT-HFD: wild-type mice fed with high-fat diet; *ASP-def*-ND: *ASP-deficient* mice fed with normal diet; *ASP-def*-HFD: *ASP-deficient* mice fed with high-fat diet; eWAT: epididymal white adipose tissue. Scale bars in (**A**) 20 μm. *ASP*: asprosin, UCP1: uncoupling protein 1, PGC1-α: PPARgamma coactivator 1, PRDM16: PRD1-BF-1-RIZ1 homologous domain-containing protein 16.
