# Supplementary material for: Angiogenesis–Browning Interplay Mediated by Asprosin-Knockout Contributes to Weight Loss in Mice with Obesity
Source: Int J Mol Sci. 2022 Dec 18;23(24):16166. doi: 10.3390/ijms232416166 (PMC9783228; doi:10.3390/ijms232416166)
Supplement: Supplementary file 1 [file ijms-23-16166-s001.zip › SI.pdf]

Supplementary Data

1. Flow chart of generating *ASP*-conditional knockout mice

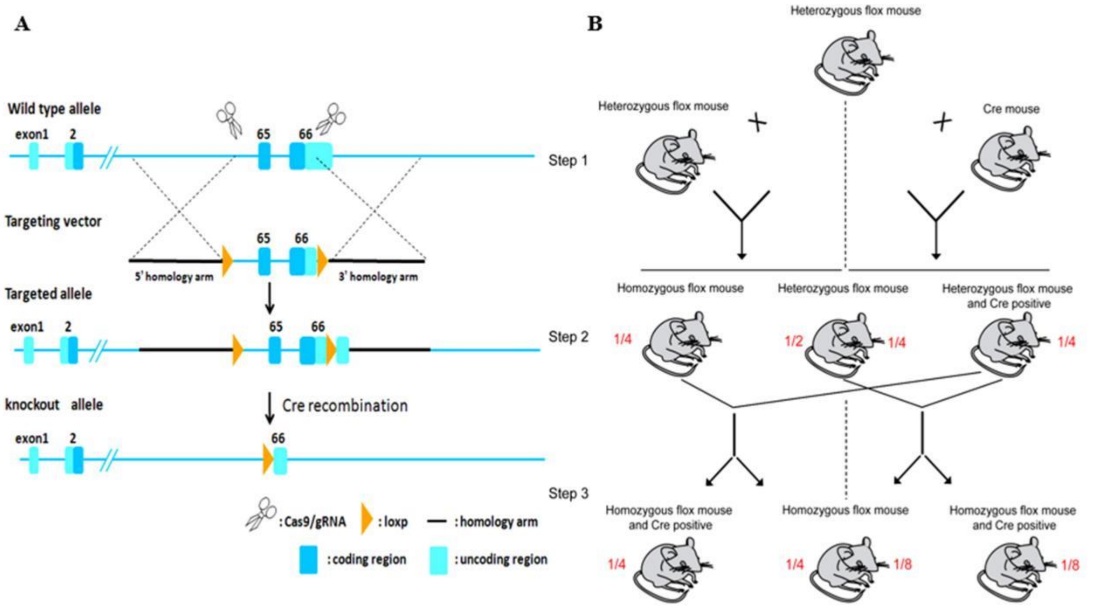

Supplementary Figure S1: Flow chart of generating *ASP*-conditional knockout mice. A: Construction flow chart of *ASP*-conditional knockout mice. B: Breed flow chart of *ASP*-conditional knockout mice.

2. Construction of *ASP*-knockout-3T3-L1 (*ASP*$^{-/-}$-3T3-L1)

ASP is 140 aa-containing peptide encoded by exon 65 and exon 66 of human profibrillin gene (*FBNI*). We designed two sgRNAs targeting *ASP* and a sgRNA of Vehicle (Figure S2B). The sgRNAs were sub-cloned to a GV392 vector (Figure S2A) which was packed to a lenti-virus vector (Lenti-CAS9-puro). The viruses carrying sgRNAs of *ASP* or Vehicle infected the 80% confluent 3T3-L1 cells. The cells resistant to puromycin were sorted out and were performed a mono-clonal culture. Subsequently, The cells were passaged more than 10 generations. Ultimately, the ASP levels were determined by western blotting and the *ASP*$^{-/-}$-3T3-L1 cell line was constructed (Figure S2C).

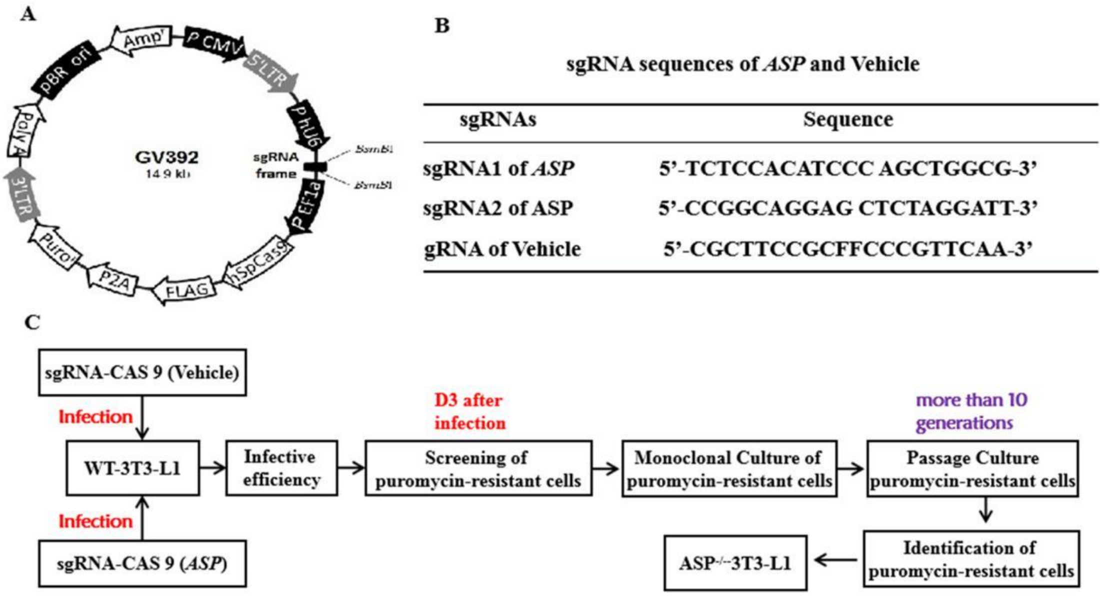

Supplementary Figure S2: Construction of *ASP*$^{-/-}$-3T3-L1cell line. A: vector map. B: sgRNA sequences of *ASP* and Vehicle. C: Flow chart of generating *ASP*$^{-/-}$-3T3-L1cell line.

3. Effect of *ASP*-CKO on PI3K/AKT signaling in aortic tissue of mice

As shown in Supplementary Figure S3, compared to WT, *ASP*-CKO strikingly increased the ratios of p-PI3K/PI3K and p-AKT/AKT in aortic tissue of mice, indicating that *ASP*-CKO activates the PI3K/AKT signaling. Moreover, *ASP*-CKO significantly increased the CD31 levels regardless of in the state of ND or HFD (Figure S1A, S1C-E), Suggesting that the increased CD31 levels might be a positive correlation with the activation of the PI3K/AKT signaling. Of course, the exact mechanism underlying the expression increase of CD31 needs to be further investigated in the future (Supplementary Figure S3)

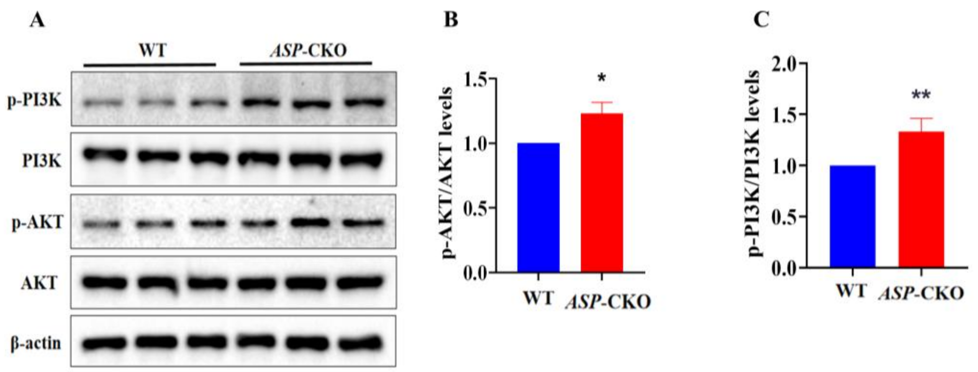

Supplementary Figure S3: Effect of *ASP*-CKO on PI3K/AKT signaling in aortic tissue of mice. Expression levels of the PI3K/AKT signaling in aortic tissue of mice (A-C). Data were presented as means ± SEM from 3 independent experiment (n=3). Unpaired two-tailed Student's t-test was used. $^{*}P<0.05$, $^{**}P<0.01$, vs WT group.

4. Effect of *ASP*-CKO on food intake in mice

As shown in Supplementary Figure S4, compared to WT mice, *ASP*-CKO mice did not show the increase of food intake, and there was no significant difference in each group (Supplementary Figure S4).

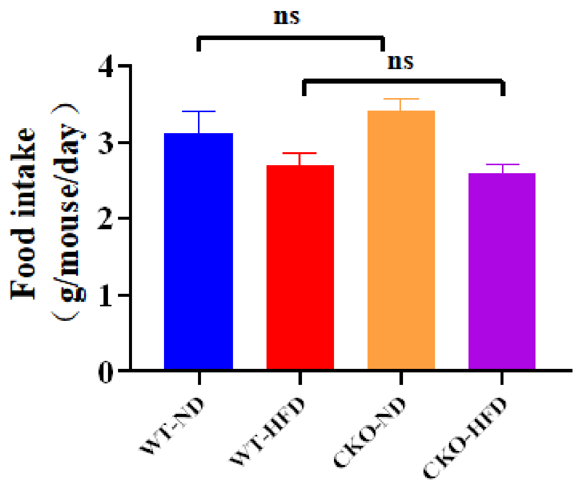

Supplementary Figure S4: Effect of *ASP*-CKO on food intake in mice. Data were presented as means ± SEM from 10 mice (n=10). One-way ANOVA was performed followed by unpaired two-tailed Student's t-test. ns: $P\geq0.05$, vs WT group.

5. Effect of *ASP*-knockout on the differentiation of 3T3-L1 preadipocytes

PPARγ and FASN are the markers of mature adipocytes. We used the markers to evaluate the the differentiation of 3T3-L1 preadipocytes. As shown in Supplementary Figure S5, no significant changes of both PPARγ and FASN levels were found in whether WT or *ASP*$^{-/-}$ group, demonstrating *ASP*-knockout did not affect the differentiation of 3T3-L1 preadipocytes (Supplementary Figure S5).

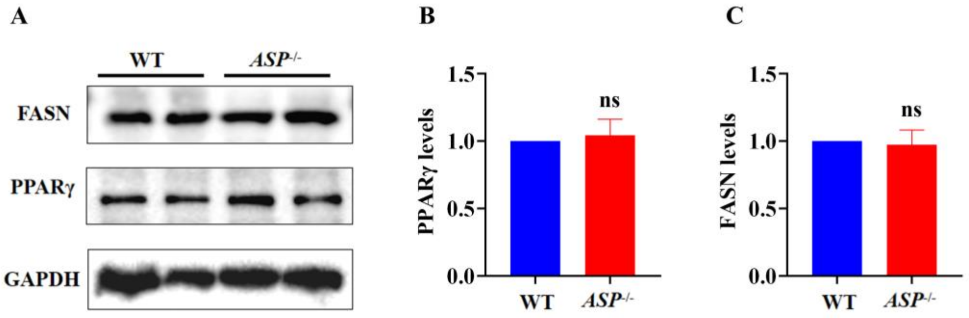

Supplementary Figure S5: Effect of *ASP*-knockout on the differentiation of 3T3-L1 preadipocytes. as means ± SEM from 3 independent experiment (n=3). Unpaired two-tailed Student's t-test was used. ns: $P \geq 0.05$, vs WT group.
